# Database of summer fish fauna sampled in river estuaries in the southern part of the Boso Peninsula, Japan

**DOI:** 10.3897/BDJ.9.e67168

**Published:** 2021-06-29

**Authors:** Rei Itsukushima, Yuichi Kano

**Affiliations:** 1 Tokyo Institute of Technology, Yokohama, Japan Tokyo Institute of Technology Yokohama Japan; 2 Kyushu University, Fukuoka, Japan Kyushu University Fukuoka Japan

## Abstract

**Background:**

River estuaries provide various ecosystem services, such as nutrient circulation, climate change mitigation, habitats and coastal defence. Information on the various taxonomic groups is collected from large-scale estuaries; however, few studies have focused on river estuaries of small and medium-sized rivers. In particular, information on river estuaries in peninsulas and islands with complex marine environments is lacking.

**New information:**

This paper provides basic information on summer fish fauna in the southern part of the Boso Peninsula, Japan. The Boso Peninsula is located at the northernmost point of where the warm current (Kuroshio) reaches and is considered to have highly endemic fish fauna. In total, 28 families, 51 species and 2,908 individuals were collected from the 27 river estuaries. The data are all accessible from the document “database_fish_estuary_boso (http://ipt.pensoft.net/manage/resource.do?r=database_fish_estuary_boso)”. Further, *Sicyopterus japonicus* and *Microphis brachyurus*, which appear in estuaries that are influenced by the Kuroshio, were confirmed. However, these species were confirmed in few of the rivers studied, highlighting the importance of habitat conservation.

## Introduction

River estuaries have complex and dynamic environments due to the influence of waves, periodic tides and mixing of freshwater and saltwater (Dyer 1997, Schroder-Adams et al. 2014). The river estuarine biological community is comprised of marine and freshwater organisms, in addition to the endemic species of brackish water (Sousa et al. 2007, Sheaves and Johnston 2008, Whitfield et al. 2012). Furthermore, the intertidal environment provides important nursery habitats for marine larvae and juvenile marine fishes (Bozeman and Dean 1980, Winkler et al. 2003, Vanalderweireldt et al. 2019). Therefore, river estuaries are especially important targets for management and conservation.

In addition, river estuaries provide various ecosystem services, such as nutrient circulation, climate change mitigation, habitats and coastal defence (Boorman 1999), estimated at $22,832/hectare/year amongst 21 biomes (Costanza et al. 1997). Humans actively use river estuaries, which results in anthropogenic effects, such as river improvement (Cohen and Carlton 1998, Edgar et al. 2000). Studies have estimated that 61% of the world’s population live in coastal areas (Alongi 1998, Bianchi 2007) and serious environmental problems, such as water degradation, disappearance of wildlife habitats and natural resource depletion, are increasing (Clark 1992, McIntyre 1995, Brown and McLachlan 2002, Kennish 2002, Howarth 2008). Information on the distribution and abundance of species is essential to conserve river estuaries where anthropogenic impacts are strong. Data regarding fish fauna in large estuaries or in the estuaries of large rivers have been collected by the National Census on River Environments (conducted by the Ministry of Land, Infrastructure, Transport and Tourism) and the National Survey on the Natural Environment (conducted by the Ministry of Environment). On the other hand, data for small and medium rivers belonging to the peninsula or islands are managed by local governments and have rarely been investigated in Japan. The lack of data has led to a shortage of knowledge about sites relevant to conservation, resulting in difficulty in determining the importance of many river estuaries.

The ocean in the southern part of the Boso Peninsula, which is the subject of this study, consists of various environments with contrasting elements, such as inner bay and open ocean, shallow and deep ocean and warm and cold currents. In particular, the Peninsula is located at the northern limit of where the Kuroshio flows along the coast of the Japanese Archipelago. As the biotas of river estuaries are strongly influenced by complex marine environments, the biota of each river is assumed to be different, although located in the same Peninsula. In this paper, we report data on fish fauna collected from 27 rivers in the southern part of the Boso Peninsula, Japan, with the aim of providing information for the conservation of the estuaries of rivers that have diverse marine environments.

## Sampling methods

### Sampling description

Habitats in half tide and spring tide belonging to one reach section (approximately 10 times the width of the river mouth) were selected as investigation sites. As it is known that the fish biomass and number of species in estuarine areas increases in summer (Shimamura and Nakamura 2001, Selleslagh et al. 2012), this study focused on the summer season or, more precisely, from 20 August to 3 October 2020. Surveys conducted throughout the year in the surf zones of outer Tokyo Bay (close to the study site) have shown that the highest number of fish species occurs in the summer, with more than 70% of the year’s total species (Arayama et al. 2002). The fishes were collected by hand nets and throwing nets at each habitat (rapid, riffle, run and pool). For each habitat, approximately 20 net casts (half mesh 5.0 mm, 14.0 m in circumference) and 30 min of sampling with a hand net (500 mm in diameter, 6 mm mesh) were conducted. The survey was conducted from 20 August 2020 to 3 October 2020. In this research, we recorded 279 occurrence data and they were identified on site according to Okamura and Amaoka (1997), Kawanabe and Mizuno (1989), Seno (2007), Toyota and Seki (2019) and Miura (2008). Of 279 occurrence data, the tissue sections (e.g. fins of the fish) of 77 specimens were preserved for future DNA analyses in absolute ethanol (-30°C) in addition to three formalin specimens. The specimens were temporarily numbered by Y. Kano's personal acronym (QUYK) and they will be deposited in official institutes (such as The Kyushu University Museum) in the future. The dataset of this paper was registered as https://ffish.asia/BosoBrackish.

## Geographic coverage

### Description

**Figure 1.**
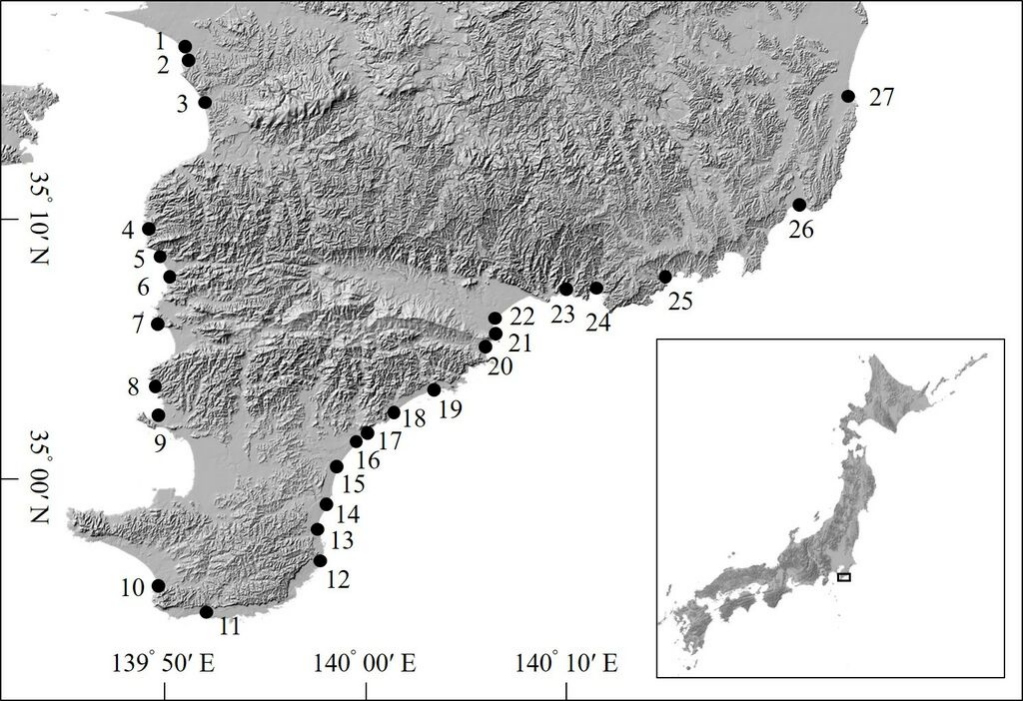
Location of the study site.

We surveyed 27 river estuaries in the southern part of the Boso Peninsula in Japan (Fig. 1). Watershed areas of investigated rivers ranged from 1.8 km^2^ to 82.0 km^2^.

### Coordinates

34.888 and 35.284 Latitude; 139.730 and 140.416 Longitude.

## Taxonomic coverage

### Description

**Figure 2.**
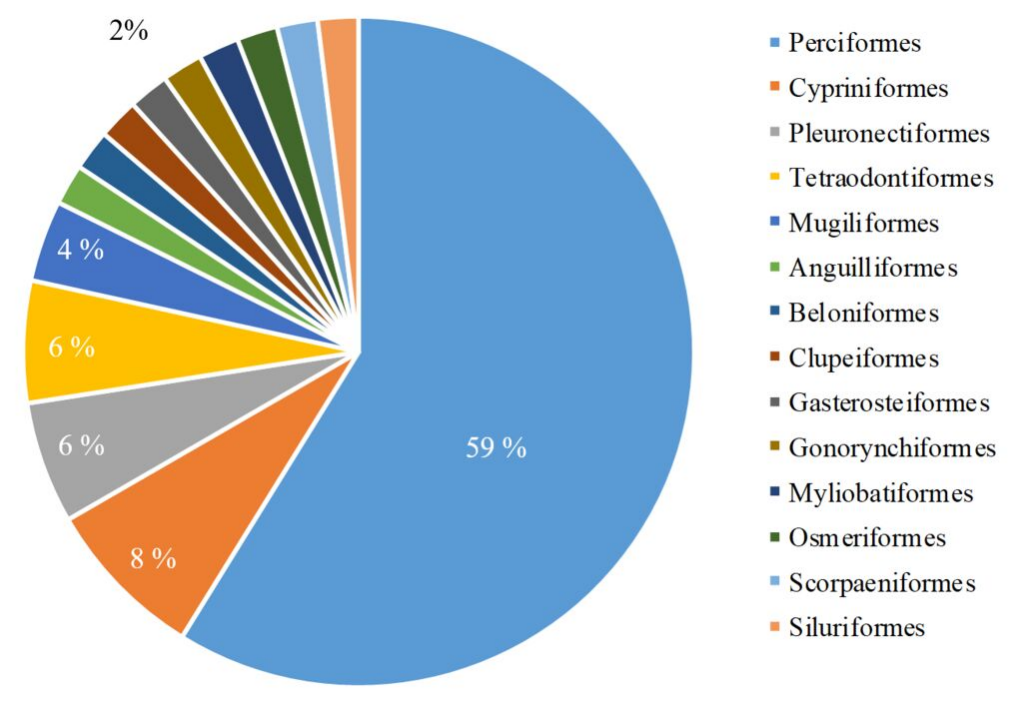
Taxonomic coverage of fish fauna (by order).

**Figure 3.**
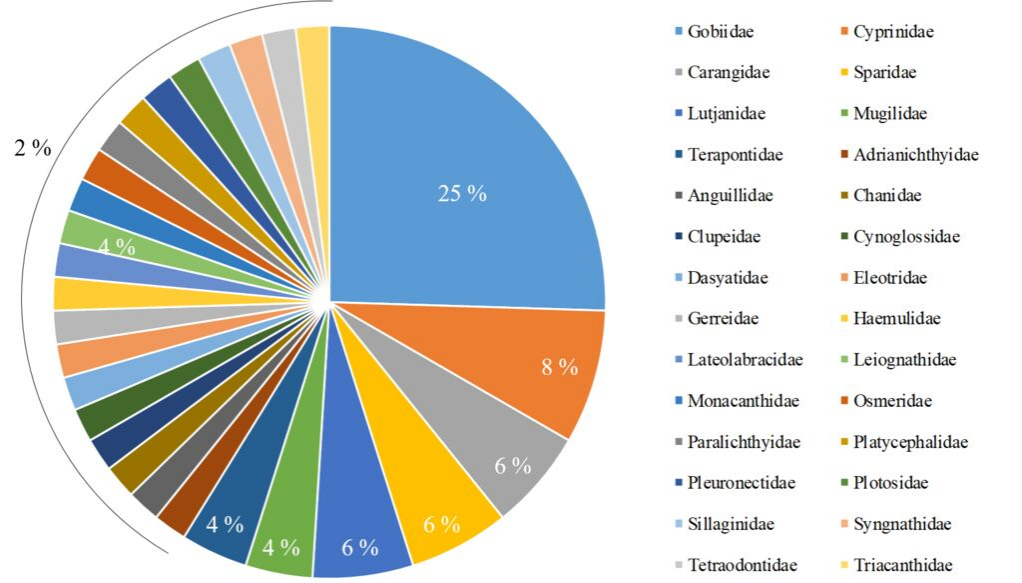
Taxonomic coverage of fish fauna (by family).

Of the fish fauna, 28 families, 51 species and 2,908 individuals were collected from the 27 river estuaries (Suppl. material 1). The Nagao River had the highest number of species (13 species) and the Oobizo River had the highest number of individuals (235 individuals). By contrast, the Kawaguchi River and the Soro River presented the lowest number of species (three species each) and the Motona River the lowest number of individuals (10 individuals). The highest number of individuals found was 1,492 of Mugil cephalus, which appeared in all target rivers. We recorded species within the following order: Perciformes (30 species), Cypriniformes (4 species), Pleuronectiformes (3 species), Tetraodontiformes (3 species), Mugiliformes (2 species), Anguilliformes (1 species), Beloniformes (1 species), Clupeiformes (1 species), Gasterosteiformes (1 species), Gonorynchiformes (1 species), Myliobatiformes (1 species), Osmeriformes (1 species), Scorpaeniformes (1 species) and Siluriformes (1 species) (Fig. 2). We recorded species from the following families: Gobiidae (13 species), Cyprinidae (4 species), Carangidae (3 species), Sparidae (3 species), Lutjanida (3 species), Mugilidae (2 species), Terapontidae (2 species), Adrianichthyidae (1 species), Anguillidae (1 species), Chanidae (1 species), Clupeidae (1 species), Cynoglossidae (1 species), Dasyatidae (1 species), Eleotridae (1 species), Gerreidae (1 species), Haemulidae (1 species), Lateolabracidae (1 species), Leiognathidae (1 species), Monacanthidae (1 species), Osmeridae (1 species), Paralichthyidae (1 species), Platycephalidae (1 species), Pleuronectidae (1 species), Plotosidae (1 species), Sillaginidae (1 species), Syngnathidae (1 species), Tetraodontidae (1 species) and Triacanthidae (1 species) (Fig. 3).

### Taxa included

**Table:** 

Rank	Scientific Name	
species	*Microphis brachyurus* (Bleeker, 1854)	
species	*Anguilla japonica* Temminck & Schlegel, 1846	
species	*Platichthys bicoloratus* (Basilewsky, 1855)	
species	*Sillago japonica* Temminck & Schlegel, 1843	
species	*Mugil cephalus* Linnaeus, 1758	
species	*Planiliza macrolepis* (Smith, 1846)	
species	*Nuchequula nuchalis* (Temminck & Schlegel, 1845)	
species	*Terapon jarbua* (Forsskål, 1775)	
species	*Rhyncopelates oxyrhynchus* (Temminck & Schlegel, 1842)	
species	*Gerres equulus* Temminck & Schlegel, 1844	
species	*Plotosus japonicus* Yoshino & Kishimoto, 2008	
species	*Paraplagusia japonica* Temminck & Schlegel, 1846	
species	*Hemitrygon akajei* (Müller & Henle, 1841)	
species	*Plectorhinchus cinctus* (Temminck & Schlegel, 1843)	
species	Platycephalus sp. sensu Nakabo & Kai, 2013	
species	*Takifugu alboplumbeus* (Richardson, 1845)	
species	*Scomberoides lysan* (Forsskål, 1775)	
species	*Caranx sexfasciatus* Quoy & Gaimard, 1825	
species	*Caranx ignobilis* (Forsskål, 1775)	
species	*Favonigobius gymnauchen* (Bleeker, 1860)	
species	*Acanthogobius flavimanus* (Temminck & Schlegel, 1845)	
species	*Acanthogobius lactipes* (Hilgendorf, 1879)	
species	*Tridentiger obscurus* (Temminck & Schlegel, 1845)	
species	*Chaenogobius annularis* Gill, 1859	
species	*Tridentiger trigonocephalus* (Gill, 1859)	
species	*Bathygobius* sp. (unidentified) Bleeker, 1878	
species	*Luciogobius* sp. (unidentified) Gill, 1859	
species	*Luciogobius guttatus* Gill, 1859	
species	*Rhinogobius nagoyae* Jordan & Seale, 1906	
species	*Sicyopterus japonicus* (Tanaka, 1909)	
species	*Tridentiger brevispinis* Katsuyama Arai & Nakamura, 1972	
species	*Rhinogobius similis* Gill, 1859	
species	*Eleotris oxycephala* Temminck & Schlegel, 1845	
species	*Lateolabrax japonicus* (Cuvier, 1828)	
species	*Stephanolepis cirrhifer* (Temminck & Schlegel, 1850)	
species	*Triacanthus biaculeatus* (Bloch, 1786)	
species	*Konosirus punctatus* (Temminck & Schlegel, 1846)	
species	*Acanthopagrus schlegelii* (Bleeker, 1854)	
species	*Rhabdosargus sarba* (Forsskål, 1775)	
species	*Acanthopagrus latus* (Houttuyn, 1782)	
species	*Lutjanus fulvus* (Forster, 1801)	
species	*Lutjanus russellii* (Bleeker, 1849)	
species	*Lutjanus argentimaculatus* (Forsskål, 1775)	
species	*Paralichthys olivaceus* (Temminck & Schlegel, 1846)	
species	*Pseudaspius hakonensis* (Günther, 1877)	
species	*Cyprinus carpio* Linnaeus, 1758	
species	*Zacco platypus* (Temminck & Schlegel, 1846)	
species	*Carassius auratus* (Linnaeus, 1758)	
species	*Chanos chanos* (Forsskål, 1775)	
species	*Oryzias latipes* (Temminck & Schlegel, 1846)	
species	*Plecoglossus altivelis* (Temminck & Schlegel, 1846)	

## Usage licence

### Usage licence

Creative Commons Public Domain Waiver (CC-Zero)

## Data resources

### Data package title

database_fish_estuary_boso

### Resource link

https://www.gbif.org/dataset/2baad33a-e52e-4789-95ad-b288607673f8

### Alternative identifiers

http://ipt.pensoft.net/resource?r=database_fish_estuary_boso

### Number of data sets

1

### Data set 1.

#### Data set name

database_fish_estuary_boso

#### Number of columns

30

#### 

**Data set 1.** 

Column label	Column description
occurrenceID	An identifier for the Occurrence.
basisOfRecord	The specific nature of the data record.
eventDate	The date-time or interval during which an Event occurred.
scientificName	The full scientific name.
kingdom	The full scientific name of the kingdom in which the taxon is classified.
phylum	The full scientific name of the phylum or division in which the taxon is classified.
class	The full scientific name of the class in which the taxon is classified.
order	The full scientific name of the order in which the taxon is classified.
family	The full scientific name of the family in which the taxon is classified.
taxonRank	The taxonomic rank of the most specific name in the scientificName as it appears in the original record.
identifiedBy	A list (concatenated and separated) of names of people, groups or organisations who assigned the Taxon to the subject.
decimalLatitude	The geographic latitude (in decimal degrees, using the spatial reference system given in geodeticDatum) of the geographic centre of a Location.
decimalLongitude	The geographic longitude (in decimal degrees, using the spatial reference system given in geodeticDatum) of the geographic centre of a Location.
geodeticDatum	The ellipsoid, geodetic datum or spatial reference system (SRS) upon which the geographic coordinates given in decimalLatitude and decimalLongitude are based.
countryCode	The standard code for the country in which the Location occurs. Recommended best practice is to use ISO 3166-1-alpha-2 country codes.
individualCount	The number of individuals represented present at the time of the Occurrence.
organismQuantity	A number or enumeration value for the quantity of organisms.
organismQuantityType	The type of quantification system used for the quantity of organisms.
habitat	A category or description of the habitat in which the Event occurred.
catalogNumber	A list (concatenated and separated) of previous or alternative fully qualified catalogue numbers or other human-used identifiers for the same Occurrence, whether in the current or any other data set or collection.
language	A language of the resource. Recommended best practice is to use a controlled vocabulary, such as RFC 4646 [RFC4646]
country	The name of the country or major administrative unit in which the Location occurs. Recommended best practice is to use a controlled vocabulary such as the Getty Thesaurus of Geographic Names.
stateProvince	The name of the next smallest administrative region than country (state, province, canton, department, region etc.) in which the Location occurs.
municipality	The full, unabbreviated name of the next smallest administrative region than county (city, municipality etc.) in which the Location occurs. Do not use this term for a nearby named place that does not contain the actual location.
locality	The specific description of the place. Less specific geographic information can be provided in other geographic terms (higherGeography, continent, country, stateProvince, county, municipality, waterBody, island, islandGroup). This term may contain information modified from the original to correct perceived errors or standardise the description.
modified	The most recent date-time on which the resource was changed. For Darwin Core, recommended best practice is to use an encoding scheme, such as ISO 8601:2004(E).
year	The four-digit year in which the Event occurred, according to the Common Era Calendar.
month	The ordinal month in which the Event occurred.
day	The integer day of the month on which the Event occurred.
locationID	An identifier for the set of location information (data associated with dcterms:Location). May be a global unique identifier or an identifier specific to the dataset.

## Additional information

Fish fauna of the Pacific Ocean side of the Japanese Archipelago has been strongly influenced by the dispersal and vicariance of the Kuroshio (Senou et al. 2006). Itsukushima (2019) classified the fish fauna of the large rivers belonging to the Japanese Archipelago and showed that rivers flowing into the Pacific Ocean had different migratory fish that appeared depending on the presence or absence of the influence of the Kuroshio and that the boundary of the classification of fish fauna is near the Boso Peninsula. The Boso Peninsula is located at the northernmost point of the Kuroshio and is considered to have highly endemic fish fauna due to its influence. As a result of this survey, appearance of *Sicyopterus japonicus* and *Microphis brachyurus*, which appear in estuaries that are influenced by the Kuroshio (Dotu and Mito 1955, Nakazato and Fujita 1986), were confirmed in the Nagao and Sugai Rivers, respectively. These two species are known to be warm-water species dependent on the Kuroshio Current, although they have been confirmed in the north of the Boso Peninsula (Hata 2020). Both rivers are located on the Pacific side of the Archipelago, near the southern tip of the Boso Peninsula and the strong influence of the Kuroshio may be the reason for the appearance of these species. These species are widely distributed in rivers influenced by the Kuroshio; however, there are few confirmed in the rivers of the Boso Peninsula and they are important as a local population. Furthermore, the distribution of fish species that are thought to be dispersed by the Kuroshio is assumed to have moved northwards due to the rise in seawater temperature caused by climate change (Yamakawa et al. 2020). In addition, the velocity of the Kuroshio is reported to increase by 30% over 100 years (Sakamoto et al. 2005), which may lead to changes in the distribution area of dispersed species and fish fauna in the Boso Peninsula. Therefore, the fish fauna data, obtained in this study, are crucial because they provide the basis for climate change impact assessments in each river.

Of the rivers surveyed, the mouths of the Soro and Shinmei Rivers were the only ones completely closed and the number of fish species were only three and five, respectively. In these two rivers, unlike the others, river mouth closure had occurred, blocking the movement between the river and the ocean. Although there is a variety of factors that degrade estuarine biota (McKinley et al. 2011, Itsukushima et al. 2019, Schulz et al. 2020), no significant differences in habitat or water quality were identified between these two rivers and the other rivers surveyed. Therefore, we concluded that mouth closure had influenced the decline of fish species. In addition to water quality degradation and salinity reduction in brackish waters (Uno et al. 2014, Watanabe et al. 2015), river mouth closure leads to fish migration impediments from marine to river habitats and disruption of spawning accretion (McDowall 1995, Kanda et al. 2009). Furthermore, the degree of river mouth closure influences the biodiversity of the system, with the number of species at its lowest in case of complete closure (Torii et al. 2011). The results of this study also indicate that the number of species was significantly reduced in completely closed river mouths, suggesting that dredging to maintain openings of river channels and other habitat protection measures are needed to improve the habitats for fishes.

This study was conducted during the summer season when the species diversity and biomass were the highest. However, several migratory species—which seasonally utilised estuarine habitats during this survey period—have not been identified and some species that utilise estuarine habitats only during winter may not have been sampled. For example, species such as *Ophieleotris* sp.1 of Akihito et al., 2013 and *Oxyurichthys lonchotus* (Lenkins, 1903) have been reported to be present in the target area (Yamakawa et al. 2018). Therefore, it is necessary to conduct surveys in each season over multiple years. This survey, however, was conducted over a wide area at the boundary of biogeography—where no data had been previously obtained—and thus presents valuable data.

### Enter subsection title

Enter subsection text

## Supplementary Material

3B423007-48EE-5398-A928-DEFEC6679E89 10.3897/BDJ.9.e67168.suppl1 Supplementary material 1 List of populations of fish species from 27 rivers in the southern part of the Boso Peninsula, Japan Data type occurrences Brief description List of populations of fish species from 27 rivers in the southern part of the Boso Peninsula, Japan File: oo_510462.xlsx https://binary.pensoft.net/file/510462 Itsukushima R and Kano Y
